# Comprehensive Analysis of the Immune and Prognostic Implication of COL6A6 in Lung Adenocarcinoma

**DOI:** 10.3389/fonc.2021.633420

**Published:** 2021-02-26

**Authors:** Yi Ma, Mantang Qiu, Haifa Guo, Haiming Chen, Jiawei Li, Xiao Li, Fan Yang

**Affiliations:** Department of Thoracic Surgery, Peking University People’s Hospital, Beijing, China

**Keywords:** lung adenocarcinoma, *COL6A6*, tumor microenvironment, prognosis, signature, nomogram

## Abstract

Collagen type VI alpha 6 chain (*COL6A6*), a novel collagen, has been considered as a tumor suppressor and therapeutic target in several tumors. However, the functional role of COL6A6 in immune cell infiltration and prognostic value in lung adenocarcinoma (LUAD) remains unknown. Here, we evaluated *COL6A6* expression and its impact on survival among LUAD patients from The Cancer Genome Atlas (TCGA) and several other databases. *COL6A6* was downregulated in LUAD tissues compared to normal tissues at both mRNA and protein levels. *COL6A6* expression was negatively associated with pathological stage, tumor stage, and lymph node metastasis. High *COL6A6* expression was a favorable prognostic factor in LUAD. Next, we explored the associations between *COL6A6* expression and immune cell infiltration. *COL6A6* expression was positively associated with the infiltration of B cells, T cells, neutrophils and dendritic cells. Additionally, the immune cell infiltration levels were associated with *COL6A6* gene copy number in LUAD. Consistently, gene set enrichment analysis showed that various immune pathways were enriched in the LUAD samples with high *COL6A6* expression, including pathways related to T cell activation and T cell receptor signaling. The impacts of *COL6A6* on immune activity were further assessed by enrichment analysis of 50 *COL6A6*-associated immunomodulators. Thereafter, using Cox regression, we identified a seven-gene risk prediction signature based on the *COL6A6*-associated immunomodulators. The resulting risk score was an independent prognostic predictor in LUAD. Receiver operating characteristic curve analysis confirmed that the seven-gene signature had good prognostic accuracy in the TCGA-LUAD cohort and a Gene Expression Omnibus dataset. Finally, we constructed a clinical nomogram to predict long-term survival probabilities, and calibration curves verified its accuracy. Our findings highlight that *COL6A6* is involved in tumor immunity, suggesting *COL6A6* may be a potential immunotherapeutic target in LUAD. The proposed seven-gene signature is a promising prognostic biomarker in LUAD.

## Introduction

Lung cancer is the leading cause of cancer-related death globally and its incidence continues to increase (1). Non-small cell lung cancer (NSCLC) accounts for 85% of lung cancer, and lung adenocarcinoma (LUAD) is the major NSCLC subtype (2). The 5-year overall survival (OS) rate of lung cancer is approximately 15%, mainly as a result of late diagnosis and the limited treatment options (3). Therefore, novel treatment strategies and biomarkers are urgently needed.

In recent years, immunotherapy has emerged as a promising strategy for various cancers, especially lung cancer (4). The success of immunotherapy highlights the importance of the tumor microenvironment (TME). Immune cell infiltration of the TME has been shown to be associated with immunotherapy outcomes and survival among patients with solid tumors (5). For instance, tumors with T cell infiltration have a favorable response to anti-PD-1/PD-L1 therapy (6). However, only about 20% of NSCLC cases respond to immunotherapy. Further exploration of the interplay between immune cell infiltration and tumor cells will provide insights into the treatment of LUAD.

Collagen type VI alpha 6 chain (*COL6A6*) is a protein-coding gene. *COL6A6* belongs to the collagen VI (*COL6*) family which acts a vital role in the extracellular matrix. *COL6A6* encodes a 2,262-amino acid protein that contains multiple von Willebrand factor domains and forms a component of the basal lamina of epithelial cells (7). This protein may regulate epithelial cell-fibronectin interactions and participate in cell adhesion (8, 9). Cell adhesion has a strong association with cancer metastasis (10). *COL6A6* overexpression significantly suppressed tumor growth and metastasis capacity in pituitary adenoma (11). The existence of COL6 family members in the extracellular matrix indicates the potential function of these proteins in the TME. In addition, *COL6A6* was found to be highly expressed in various human organs, especially in lung. However, no previous studies have reported on *COL6A6* in lung cancer.

In this study, we explored the prognostic and immune implication of *COL6A6* in LUAD. We downloaded data from The Cancer Genome Atlas (TCGA) database for detailed analysis. The underlying roles and prognostic value of *COL6A6* in LUAD were confirmed through multiple bioinformatics analyses. Further, we systematically evaluated the association between *COL6A6* and immune cell infiltration, as well as the signaling pathways regulating the *COL6A6*-mediated immune response. Moreover, we identified an immune prognostic signature using the *COL6A6*-associated immunomodulators, and we then validated its prognostic accuracy in a LUAD dataset from the Gene Expression Omnibus (GEO) database. Finally, a nomogram was established integrating the immune signature and clinical pathological features.

## Materials and Methods

### Acquisition of Gene Expression and Clinical Data

The mRNA sequencing data (HTSeq-FPKM) and associated clinical information for LUAD were downloaded from TCGA database (https://portal.gdc.cancer.gov/, up to February 23, 2020) using TCGAbiolinks R package (12), which involved 522 cases and 535 tumor tissues. Nine cases without corresponding tumor samples were eliminated. Subsequent processing excluded cases with insufficient or missing data on age, sex, race, overall survival time, local invasion, lymph node metastasis, distant metastasis, and TNM stage. Normalized gene expression data in the form of transcripts per million (TPM) were log2 (TPM+1) transformed for survival analysis. Finally, 485 cases with eligible clinical information were included in the Cox regression analysis. Moreover, 535 tumor tissues were retained to perform Cell type Identification By Estimating Relative Subsets Of RNA Transcripts (CIBERSORTx) analysis to figure out the influence of *COL6A6* expression on the immune microenvironment. Furthermore, after creating a prognostic signature using the TCGA data, it was validated using mRNA expression data and related clinical data in the GSE26939 dataset from GEO database (http://www.ncbi.nlm.nih.gov/geo/). This dataset included 116 LUAD samples and the detailed clinical data are shown in **Supplementary Table 1**. Ethical approval and informed consent were not required in this study due to the data being publicly available in the TCGA and GEO databases.

### Bioinformatics Analysis of COL6A6 Expression and Survival

Gene Expression Profiling Interactive Analysis 2 (GEPIA2) is an updated version of GEPIA for analyzing the RNA sequencing expression data of 9,736 tumors and 8,587 normal samples from the TCGA and the Genotype-Tissue Expression (GTEx) projects, using a standard processing pipeline (http://gepia2.cancer-pku.cn/#index) (13). GEPIA2 was used to conduct expression and survival analysis of *COL6A6*. The “box plot” and “stage plot” modules allowed us to perform differential expression analysis and to compare *COL6A6* expression in different pathological stages, respectively. “Survival” module was used to evaluate the correlation of COL6A6 expression with LUAD prognosis. Additionally, the expression and survival meta-analysis of *COL6A6* in LUAD was performed using the online web portal Lung Cancer Explorer (LCE) (http://lce.biohpc.swmed.edu/lungcancer/), which integrates 56 lung cancer datasets from TCGA, Gene Expression Omnibus (GEO), and other sources (14). Mutation data were retrieved from cBioPortal for Cancer Genomics (https://www.cbioportal.org/) (15).

### Validation of COL6A6 Expression in Tissues and Cell Lines

Protein expression of *COL6A6* in LUAD and normal lung tissues were evaluated based on immunohistochemistry data from the Human Protein Atlas (HPA) (https://www.proteinatlas.org/) (16). *COL6A6* expression in various LUAD cell lines were also analyzed using GENEVESTIGATOR 7.6.0, which enables analysis of deeply curated transcriptomic data from public repositories (17).

### Construction of Protein‐Protein Interaction Networks

PPI networks were constructed using the Search Tool for the Retrieval of Interacting Genes (STRING) database (version 11.0, https://string‐db.org/) (18).The minimum required interaction score was set at 0.900 (highest confidence). The results were visualized in Cytoscape software (version 3.7.2) (19). Gene Ontology (GO) annotation and Kyoto Encyclopedia of Genes and Genomes (KEGG) pathway analysis of genes in the PPI networks were performed using the clusterProfiler R package (20). The GO annotation involved biological process (BP), molecular function (MF) and cellular component (CC) annotation. P < 0.05 was set as the cut-off criterion.

### Gene Set Enrichment Analysis

GSEA was performed to identify the biological processes influenced by the genes in the prognostic signature (21). TCGA-LUAD samples with the top 25% and lowest 25% *COL6A6* expression were used as the high and low expression groups, respectively. GSEA was accomplished in GSEA software version 4.0.3 based on the Molecular Signatures Database version 7.1. C2 (curated gene sets) and C5 (GO gene sets) were searched to identify enriched KEGG pathways and GO terms associated with *COL6A6* expression. P<0.05 and false discovery rate (FDR) <0.05 were considered to indicate statistical significance.

### Associations Between COL6A6 and Tumor-Infiltrating Immune Cells

Tumor Immune Estimation Resource (TIMER) is a comprehensive resource for systematically analysis of immune infiltrates across diverse cancer types (https://cistrome.shinyapps.io/timer/) (22). The correlations between *COL6A6* expression and the abundances of six immune infiltrates (B cells, CD4+ T cells, CD8+ T cells, neutrophils, macrophages, and dendritic cells) were estimated using the “Gene” module. Additionally, the “SCNA” module was used to explore the correlations between somatic copy number alteration and the abundances of immune infiltrates.

We further determined the associations between *COL6A6* expression and the infiltration of 22 types of tumor-infiltrating lymphocytes (TILs) in LUAD using CIBERSORTx. CIBERSORTx (https://cibersortx.stanford.edu/) is the next-generation version of CIBERSORT, which supports deconvolution of bulk RNA-Seq data (23). Among the 535 LUAD tumor tissues with complete gene expression data in the TCGA database, samples with the top 25% and lowest 25% *COL6A6* expression were included as the high and low expression groups, respectively. One thousand permutations were used. P <0.05 was set as the criterion to select the immune infiltrates that may be affected by *COL6A6* expression. In addition, the correlations between *COL6A6* expression and gene markers of TILs were also analyzed using the “correlation analysis” module in GEPIA2.

### Analysis of COL6A6-Associated Immunomodulators

Immunomodulators associated with *COL6A6* were extracted from the integrated repository portal TISIDB (http://cis.hku.hk/TISIDB/) (24). This database aims to elucidate tumor and immune system interactions by integrating multiple heterogeneous data types. Immunostimulators and immunoinhibitors that were significantly correlated with *COL6A6* expression (Spearman correlation test, P<0.05) were subjected to further analysis. More specifically, the *COL6A6*-associated immunomodulators were used to build a PPI network and were subjected to KEGG and GO analyses, as mentioned above.

### Establishment of a Prognostic Signature and Survival Analysis

A prognostic immune gene signature was identified based on the *COL6A6*-associated immunomodulators. Forward stepwise variable selection was performed using the Akaike Information Criterion in Cox models (25). Based on the immune gene signature, the risk score was generated as follows: *risk score* = *β*_1_ *x*_1_ + *β*_2_ *x*_2_ + + *β_i_x_i_* where *β_i_* is the coefficient of each gene derived from the Cox regression, and *x_i_* is the expression level of each gene. Kaplan-Meier survival curve and log-rank test were used to estimate the associations of the signature and clinical features with OS. The time-dependent receiver operating characteristic (ROC) curve analysis was performed to appraise the prognostic accuracy of the risk score using the timeROC R package (26). The TCGA-LUAD dataset was used as the training dataset and the GSE26939 dataset was used as a validation dataset.

### Construction and Evaluation of a Nomogram

As a widely used predictive model, a nomogram can provide an individualized prognostic risk assessment intuitively and visually (27). Based on clinical characteristics and the risk score, we constructed a nomogram to predict the probability of 1-, 3-, and 5- year OS using the rms R package. The concordance index (C-index) was calculated to assess the predictive accuracy of the nomogram based on a bootstrap method with 1,000 replicates. Calibration curves were plotted to compare the predicted OS with actual OS rates.

### Statistical Analysis

Statistical analysis was performed in R software version 3.6.1 and IBM SPSS Statistics version 22.0. Logistic regression analysis was used to identify the associations between clinical characteristics and *COL6A6* expression. Univariate and multivariate Cox regression analyses were conducted to assess clinical factors associated with OS. P<0.05 was considered statistically significant unless otherwise indicated.

## Results

### Expression and Function of COL6A6

**Figure 1:**
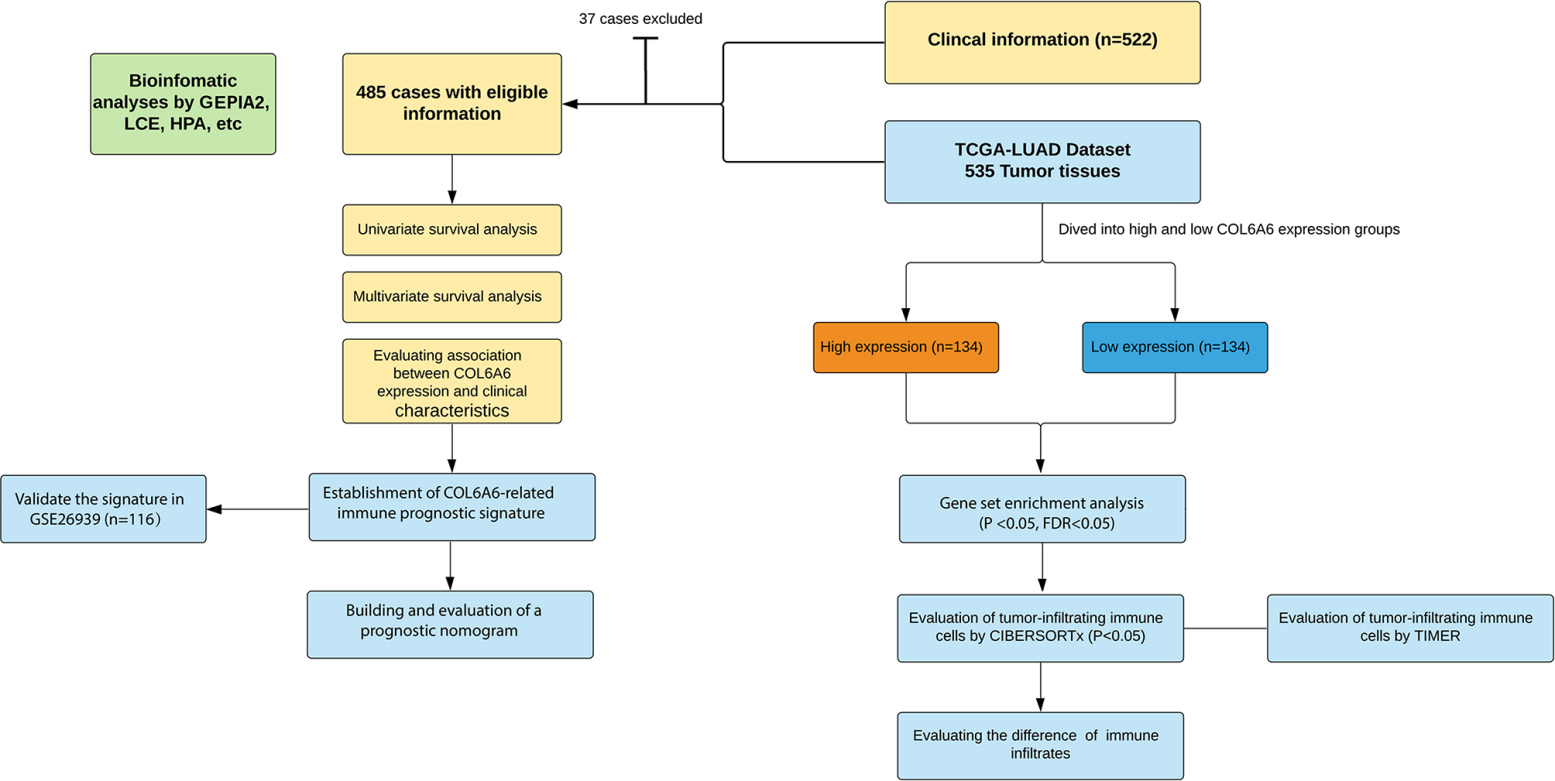
Flowchart of this study.

**Figure 2:**
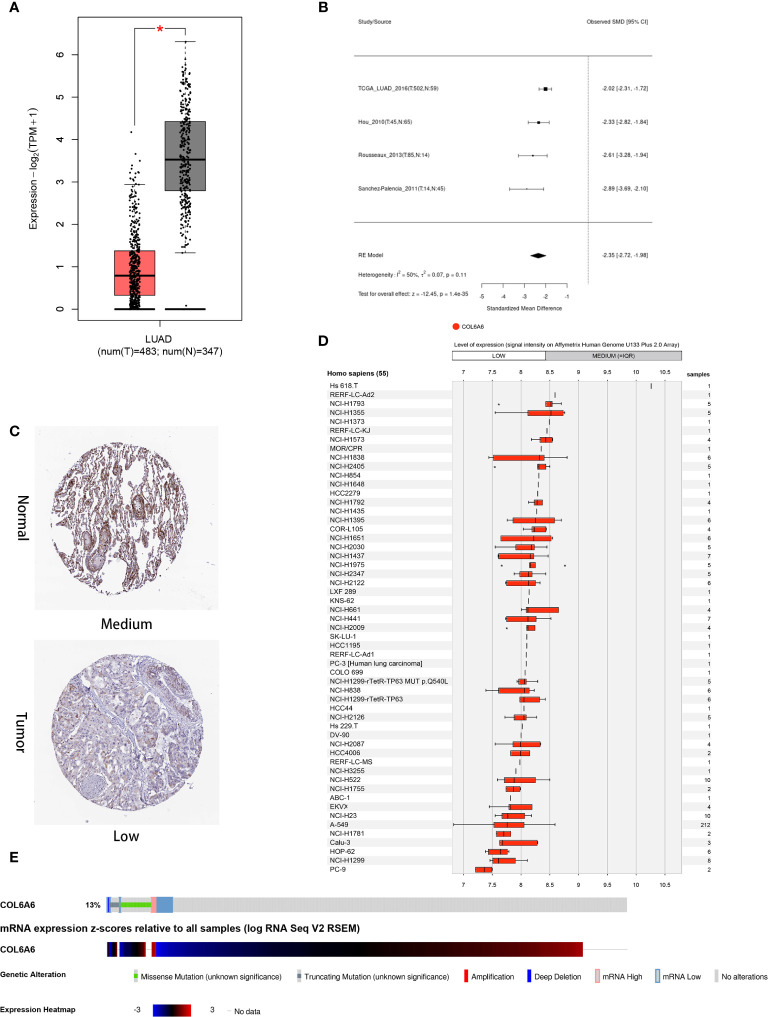
Expression and alteration of COL6A6 in lung adenocarcinoma (LUAD). **(A)** Differential COL6A6 expression in tumor tissue and matching normal tissue by GEPIA2. **(B)** Meta-analysis of COL6A6 expression in LUAD using Lung Cancer Explorer. **(C)** Representative COL6A6 protein expression in normal and LUAD tumor tissue. Data were obtained from the Human Protein Atlas. **(D)** COL6A6 expression in 55 LUAD cell lines. Data was obtained *via* GENEVESTIGATOR. **(E)** Genetic alteration and transcriptome profile of COL6A6 in LUAD. Data was obtained from cBioPortal.

**Figure 3:**
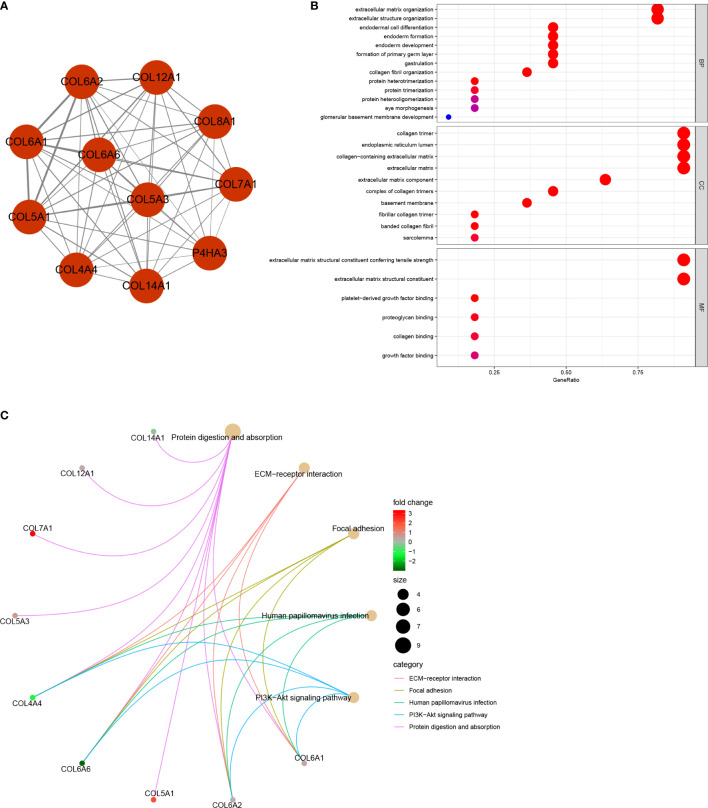
Protein‐protein interaction (PPI) network and functional annotation **(A)** PPI network of 11 genes. The line thickness represents combined scores. **(B)** 29 enriched Gene Ontology (GO) terms associated with the network. **(C)** Five enriched Kyoto Encyclopedia of Genes and Genomes (KEGG) pathways associated with the network.

This study was carried out according to the flow chart shown in **Figure 1**. According to the GEPIA2 results (**Figure 2A**), the *COL6A6* mRNA expression in LUAD tumor tissues was significantly lower than that in normal tissues (|log2(fold change) |>2, P<0.01). The differential expression was also confirmed by a meta-analysis using the LCE web portal (**Figure 2B**). Furthermore, the *COL6A6* protein expression was explored using HPA database. The typical immunohistochemistry result revealed downregulated COL6A6 expression in tumor tissues (**Figure 2C**). The protein concentration in the plasma was 490ng/L, which was detected by mass spectrometry and estimated from spectral counts in a publicly available dataset from the PeptideAtlas (**Supplementary Figure 1**). In addition, the GENEVESTIGATOR analysis showed that 92.7% (51 of 55) LUAD cell lines had low *COL6A6* expression (**Figure 2D**). According to cBioPortal, 13% of TCGA-LUAD patients had genomic *COL6A6* alterations, and missense mutation was the most common type (**Figure 2E**). As shown in **Figure 3A**, a PPI network of *COL6A6* was constructed using STRING, with the highest confidence threshold (0.900). GO annotation showed that the 11 genes in this PPI network were enriched in 29 GO terms (13 BP terms, 10 CC terms, and 6 MF terms). These terms indicated that *COL6A6* mainly plays a role in the extracellular matrix (**Figure 3B**). According to the KEGG analysis, *COL6A6* is involved in five pathways, including “Focal adhesion” and “PI3K-Akt signaling pathway” (**Figure 3C** **).**

### Survival Analysis and Prognostic Factors

**Figure 4:**
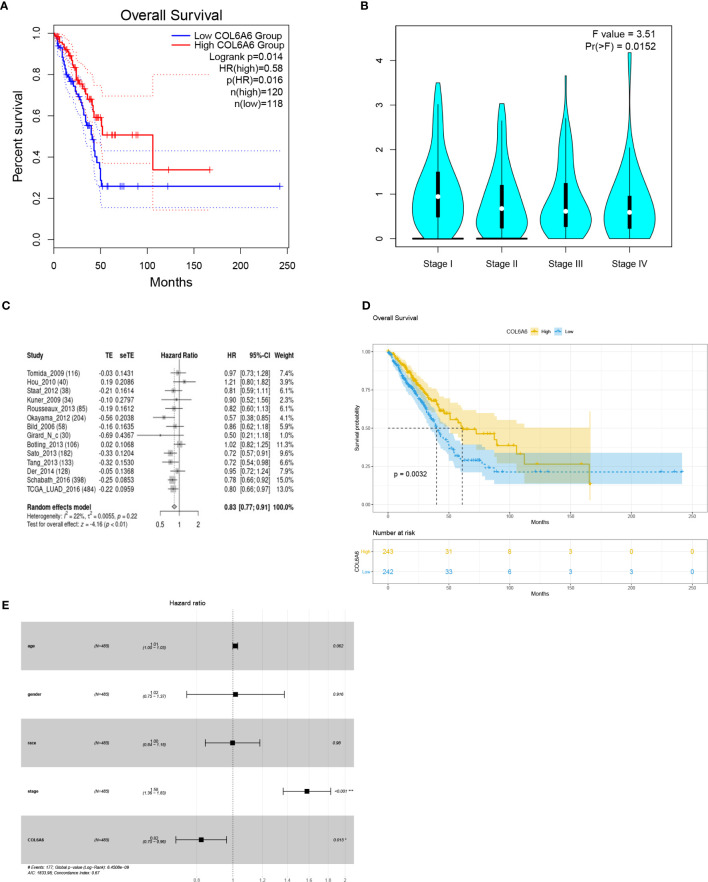
Survival analyses of COL6A6 in lung adenocarcinoma (LUAD). **(A)** Association between COL6A6 expression and survival in LUAD based on GEPIA2 (group cutoff=quartile). **(B)** Association between COL6A6 expression and pathological stage by based on GEPIA2. **(C)** Survival meta-analysis of COL6A6 in LUAD by using the LCE web portal. **(D)** Kaplan–Meier survival curve for COL6A6 expression based on the TCGA-LUAD dataset (group cutoff=median). **(E)** Multivariate Cox analysis of COL6A6 expression and clinical characteristics. Up-regulated expression of COL6A6 and low pathological stage were independent predictors of favorable prognosis.

GEPIA2 survival analysis indicated that lower *COL6A6* expression was significantly associated with poor OS (P=0.014, group cutoff=quartile; **Figure 4A**) and advanced pathological stage (P=0.015; **Figure 4B** **).** Moreover, the prognostic value of *COL6A6* was also substantiated by the survival meta-analysis using LCE web portal [hazard ratio (HR)=0.83, **Figure 4C**]. As shown in **Table 1**, we performed Cox regression analysis to identify the prognostic factors. Univariate analysis showed that pathological stage (HR=1.604, P<0.001), tumor (T) stage (HR=1.537, P<0.001), lymph node (N) stage (HR=1.668, P<0.001), and distant metastasis (M) stage (HR=1.955, P=0.02), along with *COL6A6* expression (HR=0.794, P=0.003; **Figure 4D**) were significantly associated with OS. Multivariate analysis identified up-regulated *COL6A6* expression and lower pathological stage as independent prognostic factors that predicted favorable OS (**Figure 4E**).

**Table 1:** Cox regression results of clinicopathologic variables.

Clinicopathologic variables	HR (95% CI)	P-Value
A^†^ Age Gender Race Stage T N M COL6A6	1.010 (0.994–1.025) 1.151 (0.857–1.546) 0.974 (0.823–1.154) 1.604 (1.390–1.852) 1.537 (1.281–1.844) 1.668 (1.406–1.979) 1.955 (1,110–3,445) 0.794 (0.681–0.925)	0.223 0.351 0.763 0.000 0.000 0.000 0.020 0.003
B^‡^ Stage COL6A6	1.580(1.364–1.830) 0.823(0.703–0.962)	0.000 0.015

^†^univariate cox analysis; ^‡^multivariate cox analysis.

### Association Between COL6A6 Expression and Clinical Characteristics

We investigated the mechanism underlying the effect of *COL6A6* expression in LUAD by analyzing its relationship with clinical factors. The median expression value was used to create a categorical dependent variable based on COL6A6 expression. As shown in **Table 2**, univariate logistic regression analysis revealed that *COL6A6* expression was significantly associated with age (P=0.003), pathological stage (III *vs*. I, P=0.021; III and IV *vs*. I and II, P=0.034), T stage (T2 *vs*. T1, P<0.001; T3 *vs*. T1, P=0.013; T4 *vs*. T1, P=0.012) and N stage (N1 *vs*. N0, P=0.027; N1&N2&N3 *vs*. N0, P=0.011).

**Table 2:** Association between COL6A6 expression and clinical characteristics.

Clinical characteristics	Total (N)	Odds ratio in COL6A6 expression	P-Value
Age (continuous)	494	1.028 (1.009–1.047)	0.003
Gender female *vs*. male	513	1.234 (0.873–1.750)	0.233
Race black *vs*. white others^†^ *vs*. white	439 395	0.810 (0.450–1.446) 0.315 (0.046–1.386)	0.476 0.160
Stage II *vs*. I III *vs*. I IV *vs*. I III and IV *vs*. I and II	401 359 301 511	0.723 (0.473–1.103) 0.558 (0.338–0.914) 0.602 (0.261–1.351) 0.630 (0.409–0.963)	0.133 0.021 0.222 0.034
T T2 *vs*. T1 T3 *vs*. T1 T4 *vs*. T1 T3 and T4 *vs*. T1 and T2	444 215 187 510	0.470 (0.316–0.695) 0.433 (0.222–0.833) 0.270 (0.091–0.720) 0.610 (0.357–1.029)	0.000 0.013 0.012 0.067
N N1 *vs*. N0 N2 *vs*. N0 N3 *vs*. N0 N1 and N2 and N3 *vs*. N0	432 411 339 508	0.593 (0.372–0.938) 0.649 (0.388–1.075) 0.852(0.033–21.655) 0.619 (0.426–0.897)	0.027 0.095 0.910 0.011
M M1 *vs*. M0	511	0.724 (0.318–1.600)	0.428

^†^Asian and American Indian or Alaska native.

### Gene Set Enrichment Analysis

**Figure 5:**
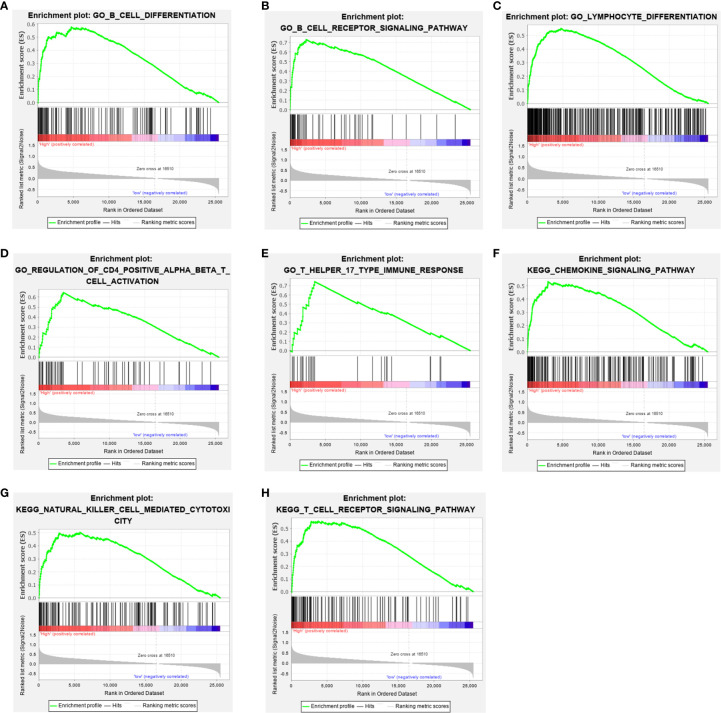
Gene set enrichment analysis (GSEA) showed that COL6A6 is involved in the tumor immune microenvironment. **(A)** B cell differentiation. **(B)** B cell receptor signaling pathway. **(C)** Lymphocyte differentiation. **(D)** Regulation of CD4 positive alpha beta T cell activation. **(E)** T Helper 17 type immune response. **(F)** Chemokine signaling pathway. **(G)** Natural killer cell mediated cytotoxicity. **(H)** T cell receptor signaling pathway.

To elucidate the role of *COL6A6* expression in the LUAD microenvironment, GSEA was used to compare the high and low expression groups. Each group included 134 samples (i.e., a quarter of the samples) from the TCGA-LUAD dataset. Various immune-related gene signatures were enriched in LUAD samples with high *COL6A6* expression, such as B cell differentiation, B cell receptor signaling pathway, T cell receptor signaling pathway, lymphocyte differentiation, regulation of T cell activation, and chemokine signaling pathway (**Figure 5** **).** These results indicate that *COL6A6* might play important roles in the tumor immune microenvironment.

### Relationship Between COL6A6 Expression and Tumor Immune Infiltrates

**Figure 6:**
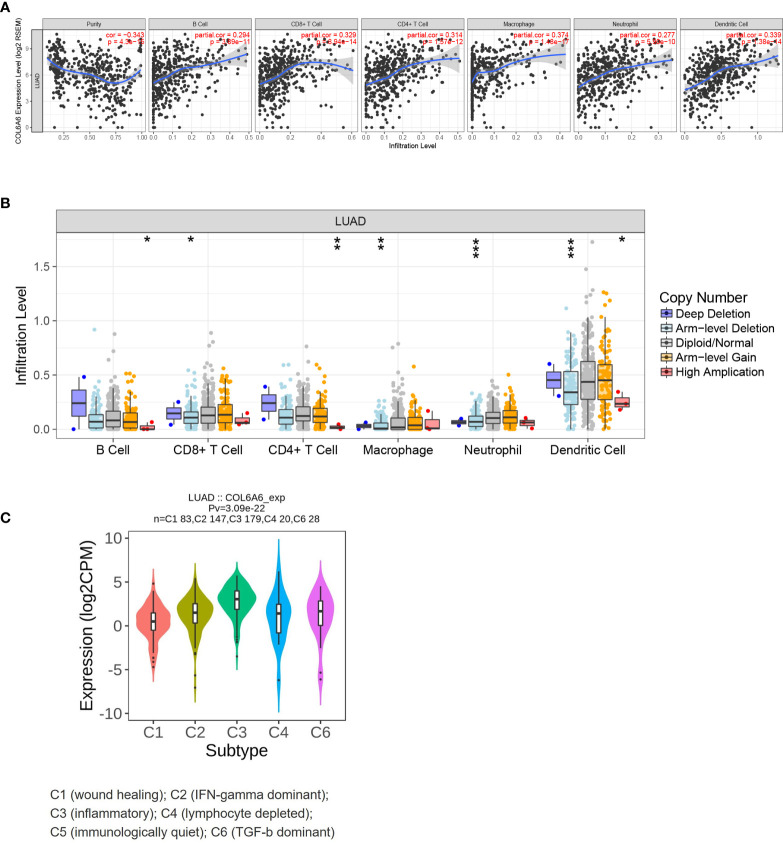
Immune relevance of COL6A6 in lung adenocarcinoma based on Tumor Immune Estimation Resource (TIMER). **(A)** Correlation between COL6A6 expression and six immune infiltrates. **(B)** Comparison of tumor infiltration levels among tumors with different COL6A6 somatic copy number alterations. **(C)** COL6A6 expression diversity in different immune subtypes according to TISIDB. *P < 0.05, **P < 0.01, ***P < 0.001.

Previous research has demonstrated that TILs act as independent predictors of survival and immunotherapy efficacy in lung cancer (28). Thus, we sought to determine whether *COL6A6* expression was related to immune cell infiltration in LUAD. The “Gene” module of TIMER was used to roughly analyze the correlations. Upregulated *COL6A6* was positively associated with the infiltration levels of all six immune cells assessed (B cells, CD4^+^ T cells, CD8^+^ T cells, neutrophils, macrophages and dendritic cells) (**Figure 6A** **, P**<0.0001). As shown in **Figure 6B**, with chromosome arm-level deletion of *COL6A6*, infiltration levels of CD8^+^ T cells (P= 0.027), macrophages (P=0.001), neutrophils (P<0.001), and dendritic cells (P<0.001) were significantly lower. In addition, we also found *COL6A6* expression varied among different immune subtypes (**Figure 6C**). *COL6A6* expression was highest in the inflammatory subtype, which indicates a better prognosis in LUAD (29).

**Figure 7:**
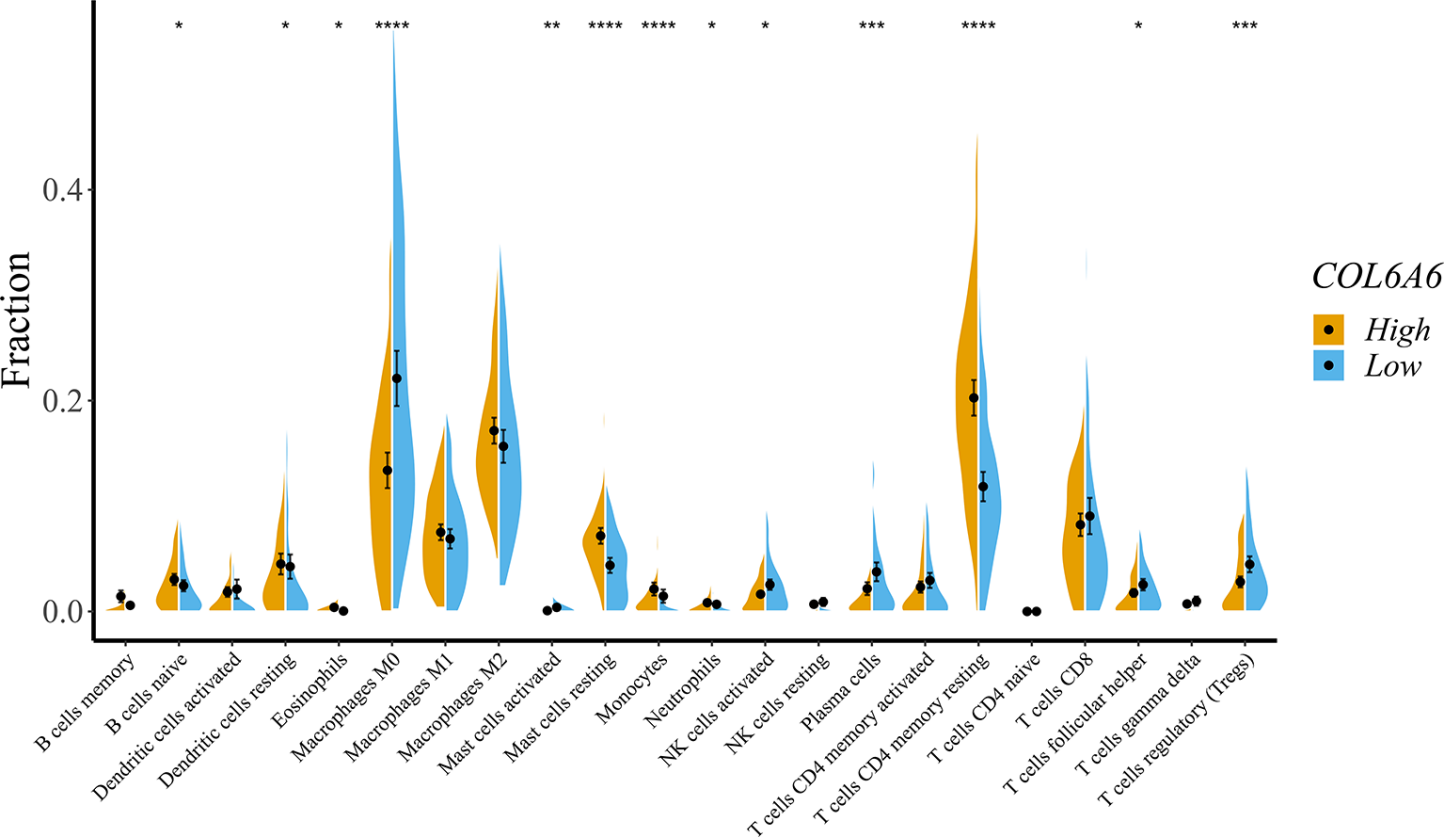
Effects on immune cell infiltration of COL6A6 in lung adenocarcinoma by CIBERSORTx. The proportions of B cells naive (P = 0.041), dendritic cells resting (P = 0.038), mast cells resting (P < 0.0001), neutrophils (P = 0.043), and T cells CD4 memory resting (P < 0.0001) were higher in the high expression group than the low expression group. In contrast, the proportion of macrophages M0 (P < 0.0001), plasma cells (P < 0.001), and Tregs (P < 0.001) were lower. *P < 0.05, **P < 0.01, ***P < 0.001, ****P < 0.0001.

To obtain a deeper understanding of the relationship between *COL6A6* expression and tumor immune infiltrates, we further determined the proportions of 22 types of TILs in LUAD using CIBERSORTx. Among the 134 TCGA-LUAD samples in each group, 125 samples in the high expression group and 107 samples in the low expression group met the screening criterion. The differences in the proportions of the 22 subpopulations of immune cells in these two groups are shown in **Figure 7**. B cells naive, dendritic cells resting, eosinophils, macrophage M0, mast cells activated, mast cells resting, monocytes, neutrophils, natural killer (NK) cells activated, plasma cells, T cells CD4 memory resting, T cells follicular helper, and T cells regulatory (Tregs) were the main immune cells correlated with *COL6A6* expression. Among them, there were higher proportions of B cells naive (P=0.041), dendritic cells resting (P=0.038), mast cells resting (P<0.0001), neutrophils (P=0.043) and T cells CD4 memory resting (P<0.0001) in the high expression group. In contrast, the proportions of macrophages M0 (P<0.0001), plasma cells (P<0.001), and Tregs (P<0.001) were lower in the high expression group. Furthermore, we analyzed the relationship between *COL6A6* expression and gene markers of various TILs, including CD8+ T cells, CD4+ T cells, B cells, neutrophils, dendritic cells and eosinophils (**Table 3**). Taking these results together, it can be concluded that *COL6A6* may play a potential role in modulating the abundance of B cells, T cells, neutrophils, and dendritic cells.

**Table 3:** Correlation between COL6A6 expression and gene markers in GEPIA2.

Immune celltypes	Gene markers	LUAD			
		Tumor		Normal	
		R	P	R	P
B cell	CD19 CD79A	0.33 0.27	1.4e−13 1.5e−09	0.053 0.15	0.69 0.26
T cell (general)	CD2 CD3E	0.41 0.43	1e−20 1e−23	0.14 0.29	0.3 0.027
CD8^+^ T cell	CD8ACD8B	0.34 0.28	1.9e−14 5.5e−10	0.21 0.055	0.11 0.68
CD4^+^ T cell	CD4	0.57	5.4e−43	0.37	0.0035
Natural killer cell	KIR2DL1 KIR2DL3 KIR2DL4 KIR3DL1 KIR3DL2 KIR3DL3 KIR2DS4	0.21 0.23 −0.025 0.25 0.18 −0.083 0.15	2.7e−06 5.8e−07 0.59 3.9e−08 9.6e−05 0.07 0.0012	0.25 0.36 0.15 0.34 0.31 0.04 0.25	0.054 0.0057 0.27 0.0078 0.015 0.76 0.054
Neutrophils	CD66b CD11b CCR7	0.37 0.42 0.52	1.9e−174 4e−22 5.7e−35	0.07 0.086 0.14	0.6 0.52 0.29
Th1	T-bet STAT4 TNF-α	0.4 0.38 0.25	2.7e−20 5.3e−18 2.3e−08	0.45 0.29 0.17	0.00032 0.028 0.21
Th2	GATA3 STAT6 STAT5A IL13	0.31 0.32 0.49 0.27	5.2e−12 1e−12 3.1e−30 9.3e−10	0.45 0.6 0.34 0.25	0.00034 5.8e−07 0.0092 0.058
Tfh	BCL6	0.26	9.4e−09	0.51	4.5e−05
Th17	STAT3 IL17A	0.29 0.13	1.4e−10 0.0045	0.54 0.12	8.5e−06 0.37
Mast cells	TPSB2 TPSAB1 CPA3 MS4A2 HDC	0.45 0.49 0.55 0.6 0.57	3.1e−25 4.3e−31 9.1e−40 5.3e−49 1.2e−42	0.33 0.42 0.45 0.5 0.43	0.011 0.001 4e−04 4.8e−05 0.00061
Eosinophils	IL5 CD125	0.17 0.43	2e−04 7.2e−23	-0.038 0.055	0.77 0.68
Monocytes	CD86 CX3CR1 CD14	0.38 0.45 0.27	6e−18 8.8e−26 1.9e−09	-0.13 0.43 0.12	0.31 6e−04 0.36
Checkpoints	PDCD1 CD274 CTLA4 LAG3 TIGIT	0.25 0.27 0.32 0.15 0.37	1.8e−08 1.2e−09 3.7e−13 0.0014 1.5e−17	0.17 0.24 0.37 0.12 0.3	0.2 7.1e−06 0.0038 0.38 0.022

GEPIA2, Gene Expression Profiling Interactive Analysis 2; LUAD, lung adenocarcinoma; Th1, T helper 1 cells; Th2, T helper 2 cells; Tfh, follicular helper T cells; Th17, T helper cell 17.

**Figure 8:**
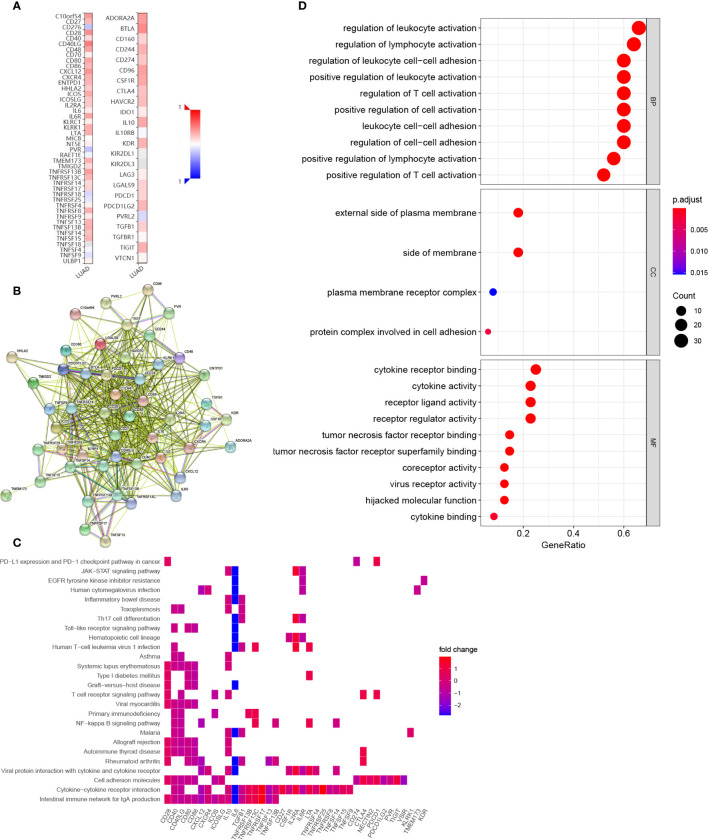
Identification and analysis of immunomodulators associated with COL6A6. **(A)** Heatmap of correlations of COL6A6 expression with immunostimulators (left panel) and immunoinhibitors (right panel) in LUAD. **(B)** Protein–protein interaction network of 50 COL6A6-associated immunomodulators in LUAD. **(C)** Gene Ontology (GO) annotation of the network. **(D)** Kyoto Encyclopedia of Genes and Genomes (KEGG) pathway analysis of the network.

We also explored the potential immune modulatory function of *COL6A6* in LUAD. We identified 33 immunostimulators (*CD27*, *VSIR*, *CD28*, *CD40*, *CD40LG*, *CD48*, *CD80*, *CD86*, *CXCL12*, *CXCR4*, *ENTPD1*, *HHLA2*, *ICOS*, *ICOSLG*, *IL2RA*, *IL6*, *IL6R*, *KLRK1*, *LTA*, *PVR*, *TMEM173*, *TMIGD2*, *TNFRSF13B*, *TNFRSF13C*, *TNFRSF14*, *TNFRSF17*, *TNFRSF25*, *TNFRSF8*, *TNFSF13*, *TNFSF13B*, *TNFSF14*, *TNFSF15*, and *TNFSF9*) and 17 immunoinhibitors (*ADORA2A*, *BTLA*, *CD160*, *CD244*, *CD274*, *CD96*, *CSF1R*, *CTLA4*, *HAVCR2*, *IL10*, *KDR*, *LGALS9*, *PDCD1*, *PDCD1LG2*, *NECTIN2*, *TGFB1*, and *TIGIT*) that were significantly associated with *COL6A6* expression in LUAD (**Figure 8A**, **Supplementary Table 2**). A PPI network was constructed based on these 50 genes (**Figure 8B**). GO and KEGG pathway enrichment analyses of these genes indicated that regulation of lymphocyte activation, regulation of T cell activation, leukocyte cell–cell adhesion, and the T cell receptor signaling pathway were related to *COL6A6*-mediated immune events (**Figures 8C, D**).

### Establishment and Validation of a Seven-Gene Signature

**Figure 9:**
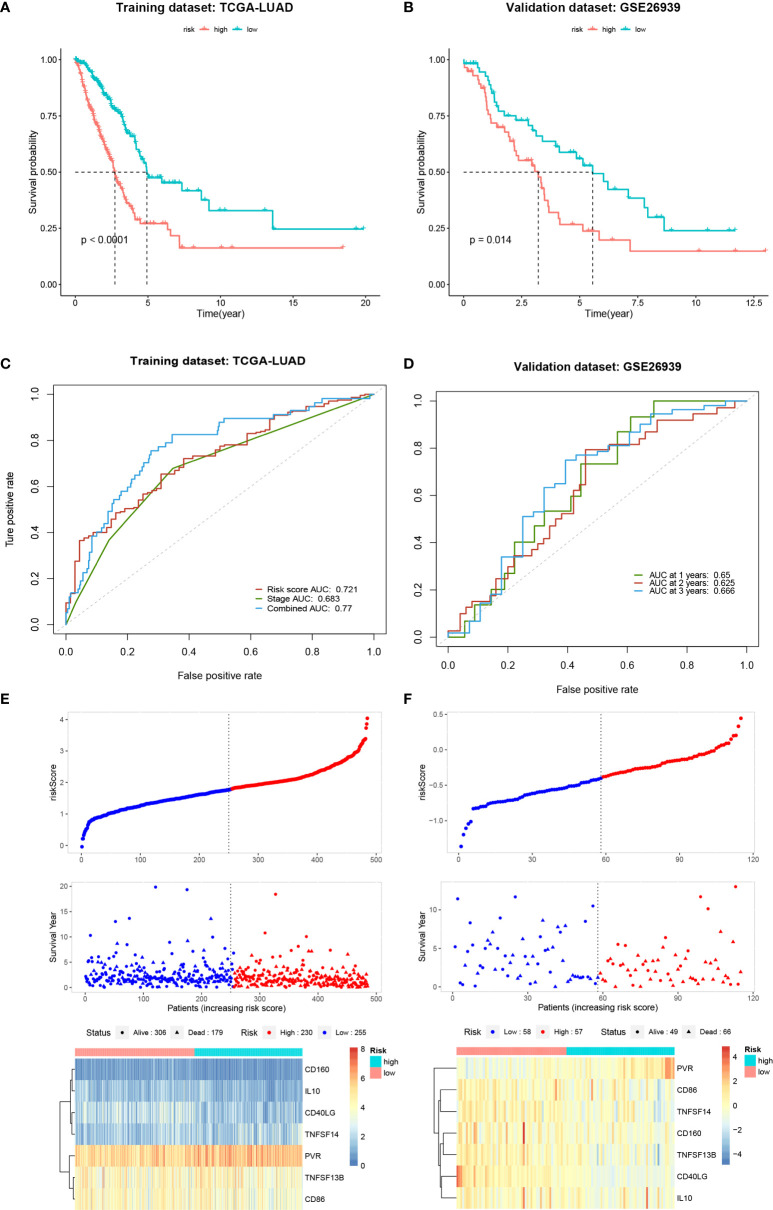
Establishment and validation of a seven-gene signature. **(A, B)** Kaplan-Meier curves of overall survival for the high and low-risk groups in the training and validation datasets. **(C, D)** Time-dependent receiver operating characteristic curves for the risk score in the training and validation datasets. **(E, F)** Distribution of risk scores, survival status and gene expression profiles for the training and validation datasets.

To determine the prognostic value of the *COL6A6*-associated immunomodulators in LUAD, we performed a stepwise Cox multivariate regression analysis. A prognostic gene signature consisting of seven genes (*CD160*, *CD40LG*, *CD86*, *IL10*, *PVR*, *TNFSF13B*, and *TNFSF14*) was identified based on the TCGA-LUAD dataset. The biological functions of the seven genes and the risk coefficients are shown in **Table 4**. Univariate Cox regression analysis was performed to evaluate the associations between these genes and OS (**Supplementary Figure 2A**). The risk scores were then calculated based on the proposed formula. Patients in the training dataset were then divided into high- and low-risk groups according to the optimal risk score cut-off. The Kaplan–Meier survival curve revealed that the survival time of the high-risk group was significantly lower (P < 0.001) (**Figure 9A**). The area under the curve (AUC) values of the risk score and pathological stage were 0.721 and 0.683, respectively. Combining the risk score and pathological stage, an AUC of 0.77 was achieved (**Figure 9C**). The risk scores, survival status distribution of the patients, and gene expression profiles related to the seven-gene signature in the training dataset are shown in **Figure 9E**. In addition, univariate Cox regression analysis showed that the risk score was significantly associated with OS (HR= 2.718, 95% CI=2.143–3.448, P<0.001). As shown in **Supplementary Figure 2B**, the risk score was an independent predictor of prognosis in the multivariate Cox regression model (HR=2.462, 95% CI=1.905–3.183, P<0.001) after adjusting for age, gender, pathological stage, T, N, and M stage. The GSE26939 dataset was used to validate the performance of the seven-gene signature. A risk score for each patient was calculated using the same method. The optimal cut-off was also determined and patients in the validation dataset were then divided into high- and low-risk groups accordingly. Consistently, the patients in the high-risk group had notably poorer prognosis (P =0.014) (**Figure 9B**). In the validation dataset, the AUCs for 1-, 3-, and 5-year OS prediction based on the gene signature were 0.65, 0.625, and 0.666, respectively (**Figure 9D**). The distribution of the risk scores and the gene expression profiles are shown in **Figure 9F**. Collectively, these results indicated that the seven-gene signature had good performance in predicting the OS of LUAD patients.

**Table 4:** Function of the genes in the prognostic signature.

Gene symbol	Full name	Function	Risk coefficient
CD160	CD160	Associated with peripheral blood NK cells and CD8 T lymphocytes with cytolytic effector activity	−0.6985597
CD40LG	CD40 ligand	Regulation of B cell function	−0.4634196
CD86	CD86	regulation of T cell activation	−0.5033796
IL10	Interleukin 10	Pleiotropic effects in immunoregulation and inflammation	−0.3230688
PVR	Poliovirus receptor	Cell adhesion and regulation of immune response	0.2712843
TNFSF13B	TNF superfamily, member 13b	Regulation of B cells proliferation and differentiation	0.8373417
TNFSF14	TNF superfamily member 14	Stimulating the activation of lymphoid cells and proliferation of T cells	0.3225482

### Construction and Evaluation of a Prognostic Nomogram

**Figure 10:**
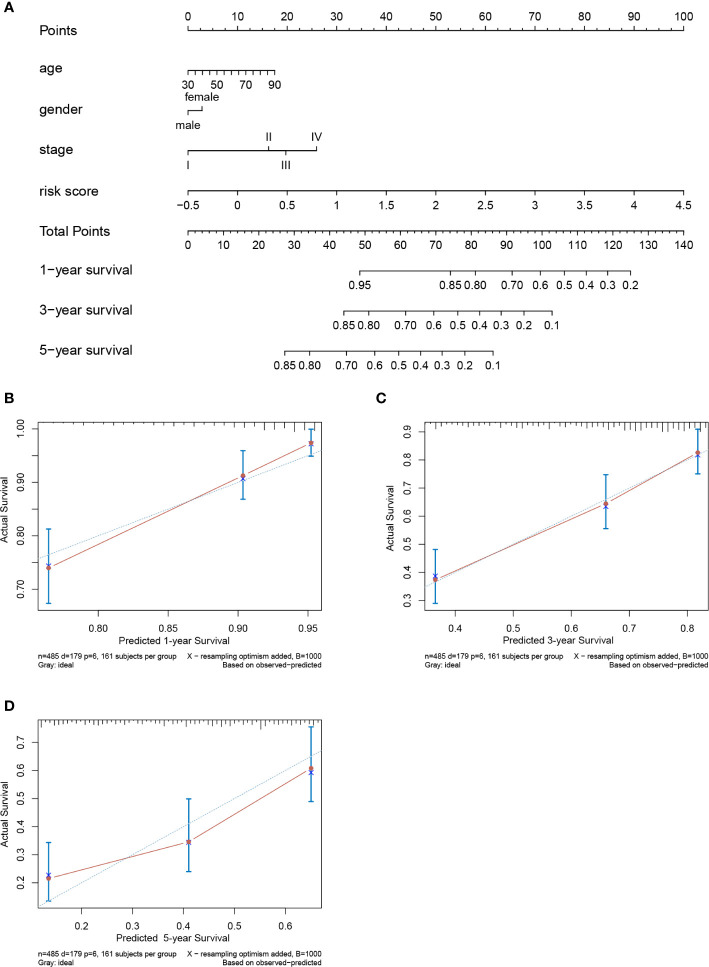
Nomogram to predict prognostic probabilities in the The Cancer Genome Atlas-lung adenocarcinoma (TCGA-LUAD) dataset. **(A)** Nomogram for predicting 1-, 3-, and 5-year overall survival (OS) of LUAD patients. **(B–D)** Calibration curves of 1-, 3-, and 5-year OS of LUAD patients. The Y axis represents the actual OS while the X axis represents nomogram predicted OS.

A prognostic nomogram to predict the 1-, 3-, and 5-year OS of LUAD patients was established based on the TCGA-LUAD dataset using Cox regression (**Figure 10A**). Risk score, age, gender and stage were features that were included in the nomogram. By calculating the score of each feature for each patient, we can predict the 1-, 3-, and 5-year OS probability, contributing to personalized precision treatment. The C-index of the prognostic nomogram was 0.723. The calibration curves further revealed that the nomogram had good performance in predicting the OS of LUAD patients (**Figures 10B–D**).

## Discussion

Lung cancer is an increasingly prevalent disease that threatens global health. Late-stage diagnosis and the limitations of classical therapies lead to a poor OS. Recent evidence suggests that immunotherapy is a powerful tool for lung cancer treatment (4–6). Therefore, the identification of novel biomarkers and promising immune-related therapeutic targets for lung cancer has become imperative in clinical practice. In this study, we found that the expression of *COL6A6* was downregulated at both mRNA and protein levels in LUAD tissues. High *COL6A6* expression was an independent predictor of a favorable prognosis. Additionally, *COL6A6* expression was significantly associated with several clinical characteristics, including pathological stage, T stage, and N stage. Moreover, *COL6A6* was found to be associated with immune cell infiltration levels and various immune pathways in LUAD. Furthermore, we identified a seven-gene risk signature based on *COL6A6*-associated immunomodulators using stepwise Cox regression model. This signature showed good predictive performance and a prognostic nomogram was constructed based on the signature for clinical application.

Previous studies revealed that *COL6A6* expression was distinctly higher in lung tissue than in other tissues (**Supplementary Figure 3**) (30, 31). However, as a new member of the *COL6* family, the role of *COL6A6* in lung cancer is unclear. We found that *COL6A6* expression in LUAD tissues was significantly lower than in normal tissues. Additionally, we found that upregulated expression of *COL6A6* was associated with a favorable prognosis. Logistic regression analysis revealed that *COL6A6* expression was related to pathological stage, T stage, and N stage. Meta-analysis effectively combines statistical strength from multiple data sets which allows for greater precision compared to when using any of the single studies. The survival meta-analysis in this study suggested that high *COL6A6* expression was a protective factor for LUAD patients. Therefore, we speculated that *COL6A6* may act as a tumor suppressor in LUAD.

Another important finding of this study was the effects of *COL6A6* on immune cell infiltration in LUAD. The TIMER analysis showed close correlations between *COL6A6* expression and abundances of B cells, CD4+ T cells, CD8+ T cells, neutrophils, macrophages, and dendritic cells. Decreased copy number may lead to reduced infiltration of CD8+ T cells, macrophages, neutrophils and dendritic cells. The above-mentioned evidence indicated the potential role of *COL6A6* in the immune microenvironment. As some immune infiltrates, such as macrophages, have been found to exist in both antitumorigenic and protumorigenic forms, we used CIBERSORTx for further analysis. Consistent with the TIMER results, we found that the proportions of several types of B cells, T cells, neutrophils, and dendritic cells were apparently increased in the high expression group while the proportions of macrophages M0 and Tregs were lower. It has been reported that the immune microenvironment is frequently associated with lung cancer outcome. T cells, especially cytotoxic and memory T cells, have been consistently shown to have positive effects on prognosis (28, 32–34). B cells, dendritic cells, and eosinophils have also been reported to be associated with a favorable prognosis (32, 33, 35). In contrast, high proportions of Tregs and macrophages M0 have been associated with a poor prognosis among LUAD patients (28, 36). Thorsson *et al*. identified six immune subtypes that span cancer tissue types and molecular subtypes, and the inflammatory subtype had the best prognosis (29). In this study, *COL6A6* expression was highest in the inflammatory immune subtype, indicating a better prognosis in LUAD; the positive impact of *COL6A6* expression prognosis among LUAD patients was consistent with the potential roles of diverse immune cell types. This further confirms the association between *COL6A6* expression and survival of LUAD patients.

Intriguingly, the GSEA results suggested that *COL6A6* was associated with differentiation and activation of several lymphocytes including B and T cells. Consistently, functional analysis of the *COL6A6*-associated immunomodulators indicated that regulation of T cell activation, leukocyte cell−cell adhesion, and T cell receptor signaling pathway were related to *COL6A6*-mediated immune events. COL6A6 participates in the cell adhesion process, and many other cell adhesion molecules, especially integrins, are known to play key roles in the regulation of immune cell infiltration into the TME (37). Integrins have various effects on T cell infiltration, including T cell recruitment into tumors, priming, and effector function (37, 38). Multiple integrin pairs, including α1β1, α2β1, α3β1, α10β1, and αvβ3 integrin, have been identified as potential binding partners for COL6 (39, 40). The PI3K-Akt pathway is another crucial pathway involved in tumor immunity. As a major pathway in tumorigenesis, its role in tumor immunity has also been reported. Okkenhaug demonstrated that the pathway contributes to the development and differentiation of B cells and T cell subsets (41). PI3K inhibition in dendritic cells enhances type I immune responses. Moreover, inhibition of the PI3K pathway enhances CD8+ T cell infiltration into tumor tissue (42). PI3K-AKT-mTOR inhibition can regulate immunosuppressive cytokine secretions, effect Treg infiltrations into tumor tissues and promote the development of memory T cells (43). The PI3K pathway may also regulate the expression of PD-L1 (44). Furthermore, COL6A6 was reported to inhibit the PI3K-Akt pathway; Long *et al*. found that *COL6A6* suppressed the growth and metastasis of pituitary adenocarcinoma by blocking the PI3K-Akt pathway (11). Together, these findings show that COL6A6 may play a pivotal role in the tumor immune microenvironment of LUAD.

Gene signatures have been widely used as prognostic predictors in various cancers (45, 46). Zhuang *et al*. analyzed M1 macrophage-related genes and identified a four-gene signature for predicting thyroid cancer prognosis (45). Li et al. analyzed the gene expression profiles of frozen tumor tissue samples from 19 public NSCLC cohorts and identified a prognostic immune-related gene signature involving 25 gene pairs (46). Their immune signature achieved moderate prognostic accuracy (C-index=0.64). In this study, we established a seven-gene signature for LUAD based on *COL6A6*-associated immunomodulators. The risk score derived from the immune gene signature was an independent predictor of LUAD prognosis. The signature had high accuracy in both the training dataset and validation dataset. Combined with clinical features, we further built a prognostic nomogram for personalized prediction, which was found to have a C-index of 0.723. Our findings may provide clinicians with a convenient and accurate way to evaluate the prognosis of patients with LUAD after surgery.

However, there are several limitations in this study. First, our study was mainly based on online databases. Nonetheless, integrating bioinformatic analyses, such as meta-analysis add strength to this study. We will further verify the expression and function of *COL6A6* as well as its correlation with immune cell infiltration in the laboratory. Second, we were unable to analyze the relationship between *COL6A6* expression and immunotherapy response, as these data in the TCGA and other databases were insufficient. Lastly, the prognostic value of the seven-gene signature should be further validated in an in-house patient population.

In summary, *COL6A6* is downregulated in LUAD tissues, while increased *COL6A6* expression predicts a favorable prognosis. *COL6A6* expression is associated with the infiltration of various immune cells, such as B cells, T cells, and dendritic cells, in LUAD. *COL6A6* may play a role in the tumor immune microenvironment of LUAD. The seven-gene signature derived from *COL6A6*-associated immunomodulators is an independent predictor of OS in LUAD. The prognostic nomogram showed good performance in predicting the OS of LUAD and might thus be beneficial for individualized treatment and medical decision making.

## Data Availability Statement

Publicly available datasets were analyzed in this study. These data can be found here: TCGA database (https://portal.gdc.cancer.gov/) and GEO database (http://www.ncbi.nlm.nih.gov/geo/).

## Author Contributions

FY, MQ, XL, and YM: study concept and design. YM, HG, HC, and JL: acquisition and analysis of the data. YM and MQ: drafting and revising of the manuscript. All authors contributed to the article and approved the submitted version.

## Funding

This work was supported by grants from the Nature Science Foundation of China (81702256 to MQ and 81802824 to XL), Natural Science Foundation of Beijing (7182169 to MQ), and Peking University People’s Hospital Scientific Research Development Funds (RDH 2020-10 to MQ).

## Conflict of Interest

The authors declare that the research was conducted in the absence of any commercial or financial relationships that could be construed as a potential conflict of interest.

## Supplementary Material

Supplementary Table 1 Clinical characteristics data of dataset GSE26939.

Supplementary Table 2 Spearman correlation analysis between expression of COL6A6 and Immunomodulators in lung adenocarcinoma from TISIDB database.

Supplementary Figure 1 COL6A6 protein concentration in plasma detected by mass spectrometry.

Supplementary Figure 2 Cox regression analyses of gene signatures and risk score. **(A)** The hazard ratios of genes integrated into the prognostic signature. **(B)** Multivariate Cox regression analysis of the risk score in LUAD regarding OS.

Supplementary Figure 3 COL6A6 expression was distinctly higher in the lung than in other tissues. **(A)** COL6A6 expression profile in different tissues according to HPA database. **(B)** COL6A6 expression profiles across all tumor samples and paired normal tissues according to GEPIA2.

## Abbreviations

ADORA2A, adenosine A2a receptor; AUC, area under the curve; BP, biological process; BTLA, B and T lymphocyte associated; CC, cellular component; CD49LG, CD40 ligand; CIBERSORTx, Cell type Identification By Estimating Relative Subsets of RNA Transcripts; C-index, concordance index; CNA, copy number alteration; COL6, collagen VI; COL6A6, collagen type VI alpha 6 chain; CSF1R, colony stimulating factor 1 receptor; CTLA4, cytotoxic T-lymphocyte associated protein 4; CXCL12, C-X-C motif chemokine ligand 12; CXCR4, C-X-C motif chemokine receptor 4; ENTPD1, ectonucleoside triphosphate diphosphohydrolase 1; FDR, false discovery rate; GEO, Gene Expression Omnibus; GEPIA, Gene Expression Profiling Interactive Analysis; GO, Gene Ontology; GSEA, gene set enrichment analysis; GTEx, The Genotype-Tissue Expression; HAVCR2, hepatitis A virus cellular receptor 2; HHLA2: HERV-H LTR-associating 2; HPA, The Human Protein Atlas; HR, hazard ratio; ICOS, inducible T cell costimulatory; ICOSLG, inducible T cell costimulator ligand; IL2RA, interleukin 2 receptor subunit alpha; IL6, interleukin 6; IL6R, interleukin 6 receptor; IL10, interleukin 10; KDR, kinase insert domain receptor; KEGG, Kyoto Encyclopedia of Genes and Genomes; KLRK1, killer cell lectin like receptor K1; LCE, Lung Cancer Explorer; LGALS9, galectin 9; LTA, lymphotoxin alpha; LUAD, lung adenocarcinoma; MF, molecular function; NECTIN2, nectin cell adhesion molecule 2; NSCLC, non-small cell lung cancer; OS, overall survival; PD-1/PDCD1, programmed cell death protein 1; PDCD1LG2, programmed cell death 1 ligand 2; PD-L1, programmed cell death protein ligand-1; PI3K-Akt-mTOR: phosphatidylinositol 3-kinase-protein kinase B-mechanistic target of rapamycin kinase; PPI, protein‐protein interactions; PVR, PVR cell adhesion molecule; ROC, receiver operating characteristic; STRING, Search Tool for the Retrieval of Interacting Genes; TCGA, The Cancer Genome Atlas; TGFB1, transforming growth factor beta 1; TIGIT; T cell immunoreceptor with Ig and ITIM domains; TILs, tumor-infiltrating lymphocytes; TIMER, Tumor Immune Estimation Resource; TNM, tumor node metastasis; TME, tumor microenvironment; TMEM173, transmembrane protein 173; TMIGD2; transmembrane and immunoglobulin domain containing 2; TNFRSF8/13B/13C/14/17/25, TNF receptor superfamily member 8/13B/13C/14/17/25; TNFSF9/13/13B/14/15, TNF receptor superfamily member 9/13/13b/14/15; TPM, transcripts per million; VSIR, V-set immunoregulatory receptor.
