# Supplementary material for: Comprehensive Analysis of the Immune and Prognostic Implication of COL6A6 in Lung Adenocarcinoma
Source: Front Oncol. 2021 Feb 26;11:633420. doi: 10.3389/fonc.2021.633420 (PMC7968342; doi:10.3389/fonc.2021.633420)
Supplement: Supplementary Table 2 — Spearman correlation analysis between expression of COL6A6 and Immunomodulators in lung adenocarcinoma from TISIDB database. [file Table_2.docx]

**Supplementary Table 2: Spearman correlation analysis between expression of COL6A6 and** [**Immunomodulator**](http://cis.hku.hk/TISIDB/browse.php?gene=CD38#inhibitor) **in lung adenocarcinoma from TISIDB database.**

| [**Immunomodulator**](http://cis.hku.hk/TISIDB/browse.php?gene=CD38#inhibitor) | **R value** | ***P* value** |
| --- | --- | --- |
| [**Immunoinhibitor**](http://cis.hku.hk/TISIDB/browse.php?gene=CD38#inhibitor) | | |
| ADORA2A | 0.383 | 0 |
| BTLA | 0.456 | 0 |
| CD160 | 0.173 | 8.7e-05 |
| CD244 | 0.307 | 1.18e-12 |
| CD274 | 0.224 | 2.87e-07 |
| CD96 | 0.372 | 2.2e-16 |
| CSF1R | 0.346 | 7.29e-16 |
| CTLA4 | 0.24 | 3.67e-08 |
| HAVCR2 | 0.264 | 1.2e-09 |
| IDO1 | 0.083 | 0.0583 |
| IL10 | 0.34 | 2.25e-15 |
| IL10RB | -0.031 | 0.487 |
| KDR | 0.293 | 1.26e-11 |
| LAG3 | 0.069 | 0.118 |
| LGALS9 | 0.178 | 5.04e-05 |
| PDCD1 | 0.174 | 6.88e-05 |
| PDCD1LG2 | 0.289 | 2.81e-11 |
| PVRL2 | -0.15 | 0.000652 |
| TGFB1 | 0.188 | 1.8e-05 |
| TGFBR1 | 0.036 | 0.411 |
| TIGIT | 0.307 | 1.35e-12 |
| VTCN1 | 0.048 | 0.273 |
| [**Immunostimulator**](http://cis.hku.hk/TISIDB/browse.php?gene=CD38#stimulator) | | |
| CD27 | 0.28 | 1.09e-10 |
| CD10orf54 | 0.397 | 0 |
| CD28 | 0.436 | 0 |
| CD40 | 0.15 | 0.000639 |
| CD40LG | 0.514 | 0 |
| CD48 | 0.367 | 0 |
| CD70 | 0.067 | 0.129 |
| CD80 | 0.355 | 7.12e-17 |
| CD86 | 0.288 | 2.86e-11 |
| CXCL12 | 0.418 | 0 |
| CXCR4 | 0.311 | 5.66e-13 |
| ENTPD1 | 0.289 | 2.47e-11 |
| HHLA2 | 0.212 | 1.16e-06 |
| ICOS | 0.336 | 5.4e-15 |
| ICOSLG | 0.218 | 6.22e-07 |
| IL2RA | 0.219 | 5.38e-07 |
| IL6 | 0.088 | 0.046 |
| IL6R | 0.366 | 8.24e-19 |
| KLRC1 | 0.073 | 0.0986 |
| KLRK1 | 0.325 | 4.34e-14 |
| LTA | 0.327 | 3.11e-14 |
| MICB | 0.015 | 0.735 |
| NT5E | 0.059 | 0.18 |
| PVR | -0.236 | 6.12e-08 |
| TMEM173 | 0.311 | 6.47e-13 |
| TMIGD2 | 0.15 | 0.000648 |
| TNFRSF13B | 0.383 | 0 |
| TNFRSF13C | 0.282 | 8e-11 |
| TNFRSF14 | 0.15 | 0.000641 |
| TNFRSF17 | 0.175 | 6.4e-05 |
| TNFRSF25 | -0.108 | 0.0137 |
| TNFRSF4 | 0.057 | 0.194 |
| TNFRSF8 | 0.316 | 2.62e-13 |
| TNFRSF9 | 0.085 | 0.053 |
| TNFSF13 | 0.297 | 6.39e-12 |
| TNFSF13B | 0.317 | 2e-13 |
| TNFSF14 | 0.252 | 7.31e-09 |
| TNFSF15 | 0.3 | 4e-12 |
| TNFSF4 | -0.038 | 0.392 |
| TNFSF9 | -0.12 | 0.00611 |
| ULBP1 | 0.062 | 0.161 |
| **P* < *0.01;* ***P* < *0.001;* ****P* < *0.0001.* | | |
