# Supplementary figures and images for: Comprehensive Analysis of the Immune and Prognostic Implication of COL6A6 in Lung Adenocarcinoma
Source: Front Oncol. 2021 Feb 26;11:633420. doi: 10.3389/fonc.2021.633420 (PMC7968342; doi:10.3389/fonc.2021.633420)

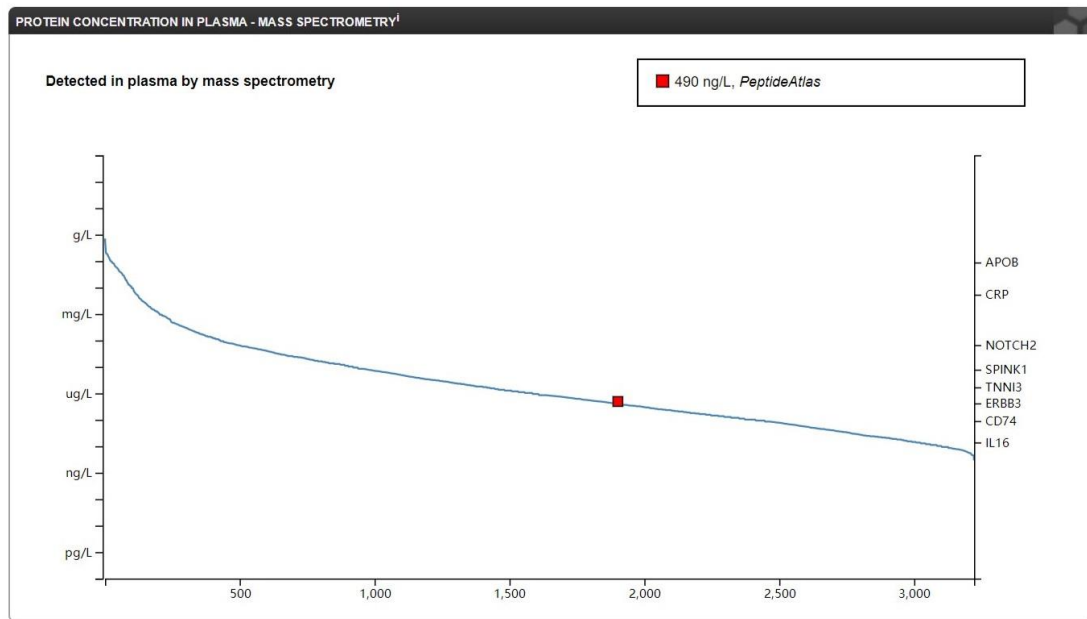

**Supplementary Figure 1.** COL6A6 protein concentration in plasma detected by mass spectrometry

Supplement: Supplementary Figure 1 — COL6A6 protein concentration in plasma detected by mass spectrometry. [file Image_1.pdf]
