# Supplementary material for: Comprehensive Analysis of the Immune and Prognostic Implication of COL6A6 in Lung Adenocarcinoma
Source: Front Oncol. 2021 Feb 26;11:633420. doi: 10.3389/fonc.2021.633420 (PMC7968342; doi:10.3389/fonc.2021.633420)
Supplement: Supplementary Figure 2 — Cox regression analyses of gene signatures and risk score. (A) The hazard ratios of genes integrated into the prognostic signature. (B) Multivariate Cox regression analysis of the risk score in LUAD regarding OS. [file Image_2.pdf]

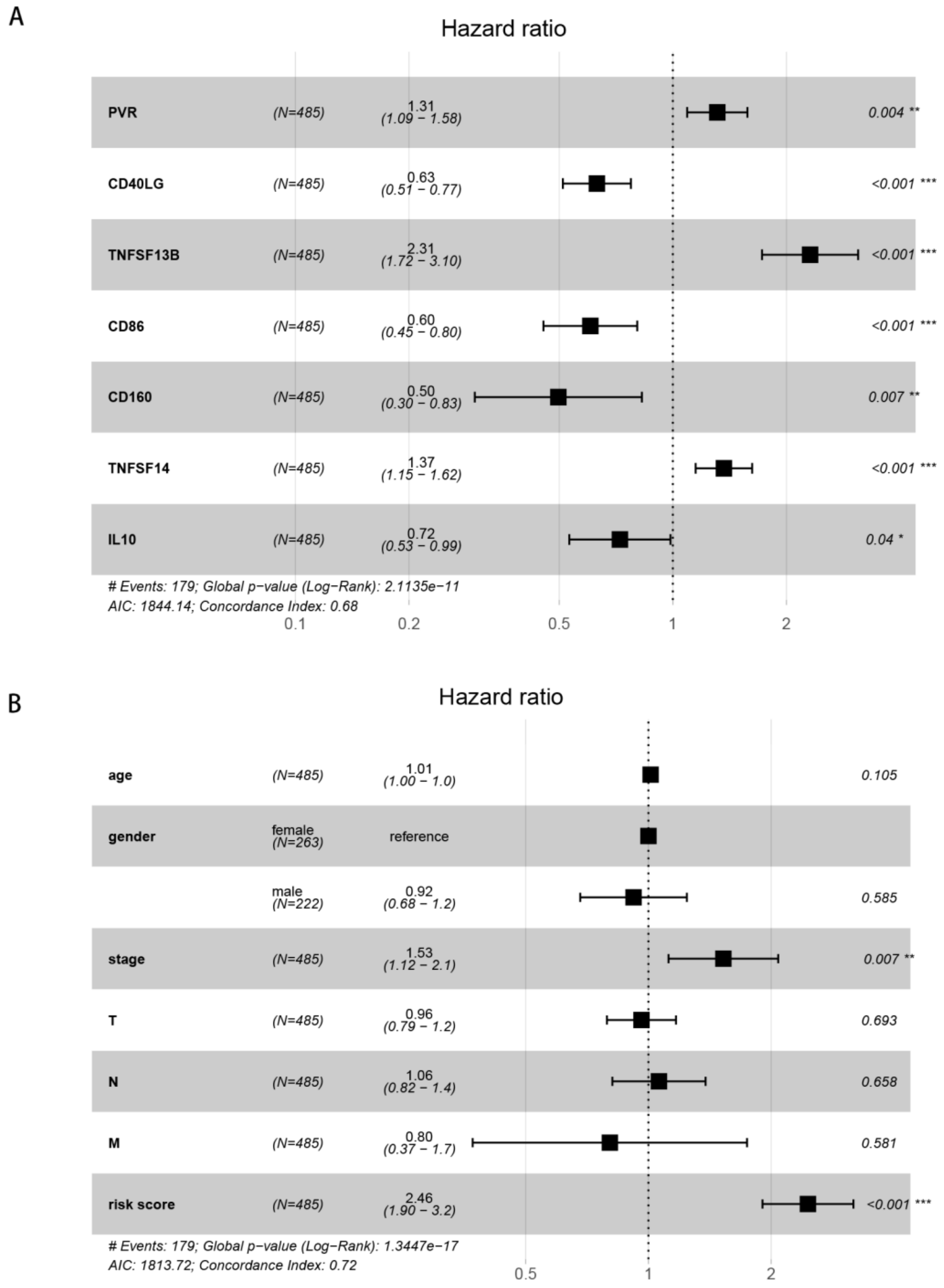

Supplementary Figure 2. Cox regression analyses of gene signatures and risk score. **(A)** The hazard ratios of genes integrated into the prognostic signature. **(B)** Multivariate Cox regression analysis of the risk score in LUAD regarding OS.
