# Supplementary material for: Comprehensive Analysis of the Immune and Prognostic Implication of COL6A6 in Lung Adenocarcinoma
Source: Front Oncol. 2021 Feb 26;11:633420. doi: 10.3389/fonc.2021.633420 (PMC7968342; doi:10.3389/fonc.2021.633420)
Supplement: Supplementary Figure 3 — COL6A6 expression was distinctly higher in the lung than in other tissues. (A) COL6A6 expression profile in different tissues according to HPA database. (B) COL6A6 expression profiles across all tumor samples and paired normal tissues according to GEPIA2. [file Image_3.pdf]

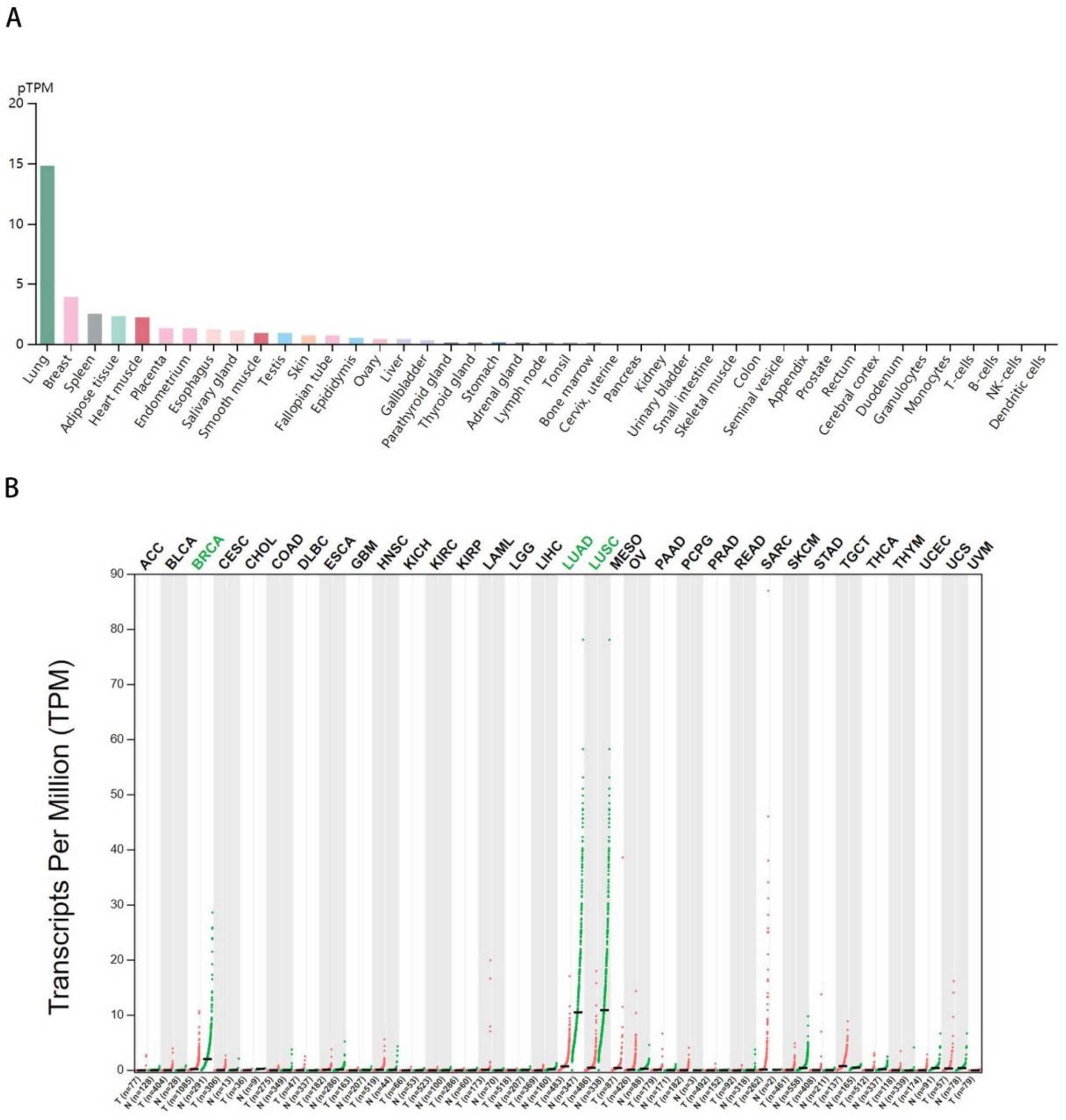

Supplementary Figure 3. COL6A6 expression was distinctly higher in the lung than in other tissues. **(A)** COL6A6 expression profile in different tissues according to HPA database. **(B)** COL6A6 expression profile across all tumor samples (red) and paired normal tissues (green) according to GEPIA2. HPA, Human Protein Atlas; GEPIA2, Gene Expression Profiling Interactive Analysis 2
